# An In Vitro Susceptibility Study of Cefotaxime-Sulbactam on Clinical Bacterial Isolates From Various Regions in India: A Comparison With Ceftriaxone-Sulbactam

**DOI:** 10.7759/cureus.36078

**Published:** 2023-03-13

**Authors:** Ajitkumar A Gondane, Dattatray B Pawar

**Affiliations:** 1 Medical Affairs, Alkem Laboratories, Mumbai, IND

**Keywords:** synergistic effects, antimicrobial resistance, minimum inhibitory concentration, β lactamase inhibitor, cephalosporins

## Abstract

Background and objective

Combining sulbactam with cefotaxime/ceftriaxone augments its antimicrobial activity against β-lactamase-producing bacteria. They are widely used as empirical treatment for many clinical infections. However, there is a scarcity of data on the susceptibility of various organisms to these antibiotics in the Indian region. In light of this, the present *in vitro* study evaluated the susceptibility of bacterial isolates to cefotaxime-sulbactam and compared it with ceftriaxone-sulbactam.

Methodology

Clinical samples with positive bacterial cultures from various laboratories in India were subjected to antibiotic sensitivity testing using *in vitro* E-test strips and disk diffusion methods to determine the minimum inhibitory concentration (MIC) and zone of inhibition (ZOI), respectively. MIC_50_ and MIC_90_ values were determined along with the measurement of the ZOI for the effectiveness of antibiotics. Interpretations of MIC and ZOI values were made as per the criteria set by the Clinical and Laboratory Standards Institute (CLSI) guidelines to estimate the proportion of sensitive organisms.

Results

Among 400 clinical isolates evaluated, *Escherichia coli (E. coli) *(47.75%) was the most common organism isolated followed by *Klebsiella* (26%), *Salmonella* (7.75%), *Proteus* (3.8%), and *Acinetobacter* (2.8%). The mean ZOI was found significantly higher for *E. coli, Klebsiella, *and* Salmonella* in the cefotaxime-sulbactam group than in the ceftriaxone-sulbactam group. MIC_50_ values for *E. coli* and *Klebsiella* were 0.25 and 0.19 µg/ml, respectively in the cefotaxime-sulbactam group as compared to 0.38 and 0.25 µg/ml, respectively for ceftriaxone-sulbactam. The proportion of sensitive isolates was also higher in the cefotaxime-sulbactam group for *E. coli, Klebsiella*, and *Salmonella*.

Conclusions

The *in vitro* effect of cefotaxime-sulbactam on organisms is similar to that of ceftriaxone-sulbactam in terms of MIC, ZOI, and proportion of sensitivity based on our study involving clinical isolates from various parts of India. Cefotaxime-sulbactam may be preferred in the empirical management of various clinical infections.

## Introduction

Cefotaxime and ceftriaxone are "third generation" semi-synthetic cephalosporins administered intravenously or intramuscularly for a variety of clinical infections like lower respiratory tract infections, kidney and urinary tract infections, skin and soft tissue infections, genital infections, intra-abdominal infections (including peritonitis), acute meningitis, sepsis, and endocarditis either alone or in combination with another suitable antibiotic [1,2]. Both of them inhibit bacterial cell wall synthesis by interfering with the transpeptidation step of cell wall synthesis after binding to one or more of the penicillin-binding proteins (PBPs). They have a broad spectrum of activity against various Gram-positive and Gram-negative aerobic and anaerobic bacteria [1-3]. Ceftriaxone is more protein-bound as compared to cefotaxime (95% vs. 35%), which may limit the *in vitro* activity of ceftriaxone, which in turn may affect its *in vivo* activity [4].

According to the National Treatment Guidelines for Antimicrobial Use in Infectious Diseases, published by the National Centre for Disease Control, Government of India, cefotaxime has been recommended as an empirical/first-line therapy for the treatment of a variety of severe infections, which has led to its widespread use in the country [5].

There have been reports of the emergence of resistance to cefotaxime and ceftriaxone, mainly with Gram-negative organisms like *Escherichia coli (E. coli), Klebsiella, Salmonella, *and* Enterobacter cloacae* and also to some extent with Gram-positive organisms like *Staphylococcus aureus* and *Streptococcus pneumoniae* [6-8]. In a study conducted by Kaistha et al. on neonatal septicemia, various Gram-negative organisms were found to be highly resistant to cefotaxime (34%-86%), ceftriaxone (57%-80%), and ceftazidime (33%-62%) [9]. The development of resistance may be due to the production of extended-spectrum β-lactamases (ESBLs) by organisms, which can destroy the antibiotic before they exert their action; induction, and/or constitutive expression of AmpC β-lactamases; reduced outer membrane permeability; efflux pump mechanisms or modification of target enzymes (altered PBPs) [10,11]. More than one of these mechanisms may coexist in a single bacterium, but the production of β-lactamase is the main mechanism of resistance [10].

Sulbactam is an irreversible inhibitor of β-lactamase that prevents the destruction of β-lactam antibiotics [12]. The addition of sulbactam to cefotaxime/ceftriaxone augments their antimicrobial activity against β-lactamase-producing bacteria and helps in overcoming the issue of resistance [12]. A combination of cephalosporins with β-lactamase inhibitors is widely prescribed by clinicians for a variety of clinical infections in India. In a study by Naveen et al., 12.6% of total prescriptions for cephalosporins in medicine and surgery inpatient departments involved a combination of cephalosporins with β-lactamase inhibitors [13]. As the reports of resistance to such combinations are not readily available in the literature, it becomes important to assess and understand the current sensitivity pattern of these drugs against the organisms for gaining further guidance on their clinical application. The data regarding the susceptibility of bacterial isolates to such widely used antibiotics are very scarce in the Indian region. Hence, the present *in vitro* study was designed to evaluate the sensitivity pattern of the widely utilized combination “cefotaxime-sulbactam” to various clinical isolates and compare it with that of “ceftriaxone-sulbactam”.

## Materials and methods

Study setting

The present active-controlled *in vitro* study involved 400 clinical samples obtained from patients (both outpatients and inpatients) presenting with various bacterial infections (e.g., urinary tract infection, lower respiratory tract infection, skin and soft tissue infection, sepsis, genital infection, intra-abdominal infection, and acute meningitis) wherein cefotaxime-sulbactam is routinely indicated. The patients' identities were not disclosed directly or indirectly in this study. Culture-positive samples across four microbiology laboratories in the East (Bhubaneshwar), West (Ahmedabad), North (Delhi), and South (Srikakulam) of India were collected and analyzed. The Institutional Review Board/Independent Ethics Committee (IRB/IEC) of each trial center reviewed and approved the study protocol and required documents.

Clinical samples of treatment-naïve patients suffering from targeted clinical infections (lower respiratory tract infection, urinary tract infection, skin and soft tissue infection, abdominal infection, genital infection, meningitis, sepsis, and endocarditis) for whom bacterial culture was positive were included for antibiotic sensitivity testing using the E-test strip method. Patients who had taken antibiotics, antiviral agents, or interferon therapy in the five days prior to the collection of the sample were excluded. Information about the probable diagnosis was collected from the request form. Organisms were isolated using standard culture media and inoculum methods. Morphological and biochemical tests for the identification of isolated organisms were performed using the standard procedure described by Cheesbrough [14]. All the positive samples meeting inclusion/exclusion criteria were tested for antibiotic sensitivity using the E-test strip and disk diffusion methods. The E-test strip method was used to determine the minimum inhibitory concentration (MIC), whereas the disk diffusion method was used for assessing the zone of inhibition (ZOI). Cefotaxime-sulbactam and ceftriaxone-sulbactam Ezy MIC^TM^ strips manufactured by HIMEDIA, which can measure MICs from 0.016 μg/ml to 256 μg/ml, were used at all the study sites. Similarly, all the sites used the disks (cefotaxime-sulbactam and ceftriaxone-sulbactam) manufactured by HIMEDIA. E-test strips and disks of the same batches were procured centrally and provided to all the study sites.

Determining MIC by E-test strip method [15]

Ezy MIC™ strips were used for determining the antibiotic susceptibility of organisms. E-test strip method provides quantitative measurement of MICs in a simple and easily reproducible manner. Ezy MIC™ is a porous paper strip coated with antibiotics and is thin and inert. It contains μg/ml scale over both sides of the strip for MIC reading. There is a maximum concentration of antibiotic at one end and a minimum at the other end with an exponential gradient that covers 15 two-fold dilutions range on the strip. When applied to the medium, a gradual and effective transfer of the preformed antibiotic gradient occurs from the strip into the agar medium. An ellipse centered at the strip suggests bacterial growth inhibition. The scale at the intersecting of the ellipse edge with the strip represents the MIC value. The E-test strip was applied to an inoculated agar surface and incubated in an inverted position overnight after 15 minutes of placing the strip under 37 °C. The E-test strip was observed for a symmetrical inhibition ellipse after bacterial growth became visible. The MIC value was read in terms of μg/ml from the scale where the ellipse edge was intersecting the E-test strip.

Determining ZOI using the disk diffusion method

For determining the ZOI, the disk diffusion method was used as per Bauer et al. [16]. A 6-mm filter paper disk impregnated with a known concentration of an antimicrobial compound was placed on the agar plate. Two disks were used in one agar plate with a distance of 5 cm. The complete contact with the agar surface of the disks was confirmed and agar plates were incubated in an inverted position overnight after drying for approximately 10-15 minutes under 37 °C. The disk was observed for a clear zone and the diameter of the ZOI surrounding the antibiotic disk was measured using a ruler or caliper and correlated with the standards in the Clinical and Laboratory Standards Institute (CLSI) guidelines to determine whether the microorganism was sensitive or resistant to the antibiotic [17].

Assessment of efficacy

Determining MIC_50_ and MIC_90_ [18]

The MIC_50_ was considered as the inhibitory concentration of antibiotic that had inhibited ≥50% of the isolates in a test population whereas the MIC_90_ was the value at which ≥90% of the strains within a test population were inhibited. Values of MICs were obtained with “n” test strains and the values y1, y2. . .yn representing a graded series of MICs starting with the lowest value; the MIC_50_ was the value at position n x 0.5 for an even number of test strains and (n+1) x 0.5 for an odd number of test strains. MIC_90_ was calculated accordingly, using n x 0.9.

Interpretation of MIC and ZOI

As per CLSI criteria (M100 2022), the organisms were classified as sensitive or resistant [17]. Intermediate organisms were considered resistant in the present study.

Statistical analysis

Descriptive statistics were used for expressing individual variables. Diagnosis, samples, and organisms were presented as proportions. MIC was presented as the geometric mean and ZOI as the arithmetic means along with a 95% confidence interval (CI). MIC and ZOI were compared between the two groups using an unpaired t-test. Proportions of sensitive organisms between both groups were compared using the Chi-square test. The statistical significance was set at 5%.

## Results

A total of 400 clinical isolates from urine (42.75%), pus (17.5%), sputum (15.75%), blood (12.75%), endotracheal secretion (5%), and endocervical secretion (3.75%) from various regions of India were analyzed for MIC and ZOI using E-test strip and disk diffusion methods, respectively (Table 1). Clinical isolates were collected from patients with urinary tract infection (42.75%), lower respiratory tract infection (20.75%), skin and soft tissue infection (16.75%), sepsis (12.75%), genital infection (3.75%), intra-abdominal infection (2%), and acute meningitis (1.3%).

**Table 1:** Sample disposition in the study (n=400)

Sample type	N	%
Urine	171	42.8
Sputum	63	15.8
Blood	51	12.8
Pus	70	17.5
Endotracheal secretion	20	5.0
Endocervical swab	15	3.8
Cerebrospinal fluid	5	1.3
Serous fluid	2	0.5
Stool	2	0.5
Tissue	1	0.3

The organisms isolated from culture methods after inoculation of samples are presented in Table 2. Aggregating all types of infections, *E. coli* (47.8%) was the most common organism isolated followed by *Klebsiella* (26%) and *Salmonella *(7.8%).

**Table 2:** Organisms isolated from samples (n=400)

Organism	N	%
E. coli	191	47.8
Klebsiella	104	26.0
Salmonella	31	7.8
Proteus mirabilis	15	3.8
Acinetobacter baumannii	11	2.8
Staphylococcus	10	2.5
Enterobacter cloacae	10	2.5
Morganella morganii	9	2.3
Citrobacter koseri	8	2.0
Pseudomonas aeruginosa	7	1.8
Serratia marcescens	4	1.0

Efficacy analysis

As shown in Table 3, MIC was found comparable between cefotaxime-sulbactam and ceftriaxone-sulbactam for isolated organisms.

**Table 3:** Comparison of MIC (geometric mean) between cefotaxime-sulbactam and ceftriaxone-sulbactam for isolated organisms *Unpaired t-test MIC: minimum inhibitory concentration

Organisms	Cefotaxime-sulbactam, geometric mean (95% CI)	Ceftriaxone-sulbactam, geometric mean (95% CI)	P-value*
*E. coli* (n=191)	0.22 (0.16 to 0.30)	0.29 (0.21 to 0.39)	0.22
*Klebsiella* (n=104)	0.27 (0.17 to 0.44)	0.40 (0.25 to 0.65)	0.24
*Salmonella* (n=31)	0.07 (0.04 to 0.10)	0.08 (0.05 to 0.11)	0.072
*Proteus mirabilis* (n=15)	0.04 (0.02 to 0.07)	0.04 (0.02 to 0.08)	0.82
*Acinetobacter baumannii* (n=11)	22.29 (2.6 to 192.5)	33.76 (4.8 to 238.3)	0.75
*Staphylococcus* (n=10)	0.87 (0.12 to 6.20)	0.95 (0.12 to 7.34)	0.95
*Enterobacter cloacae* (n=10)	1.12 (0.14 to 8.9)	1.18 (0.16 to 9.01)	0.97
*Morganella morganii* (n=9)	0.13 (0.04 to 0.45)	0.14 (0.03 to 0.59)	0.92
*Citrobacter koseri* (n=8)	0.13 (0.03 to 0.60)	0.19 (0.04 to 0.89)	0.72
*Pseudomonas aeruginosa* (n=7)	74.9 (15.2 to 368.9)	111.3 (25.5 to 285.8)	0.66
*Serratia marcescens* (n=4)	0.05 (0.03 to 0.11)	0.09 (0.04 to 0.19)	0.17

**Figure 1:**
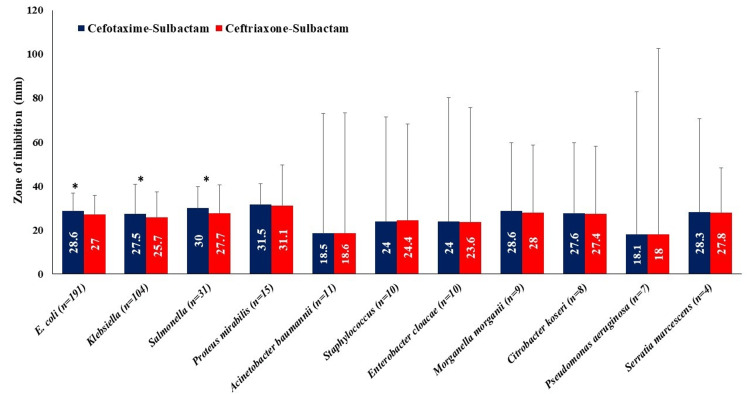
Comparisons of the mean ZOI between cefotaxime-sulbactam and ceftriaxone-sulbactam for isolated organisms *P<0.05 using unpaired t-test Data are presented as mean (SD) ZOI: zone of inhibition

The mean ZOI was found significantly higher for *E. coli, Klebsiella*, and *Salmonella* with cefotaxime-sulbactam as compared to ceftriaxone-sulbactam (p<0.05; Figure 1).

**Figure 2:**
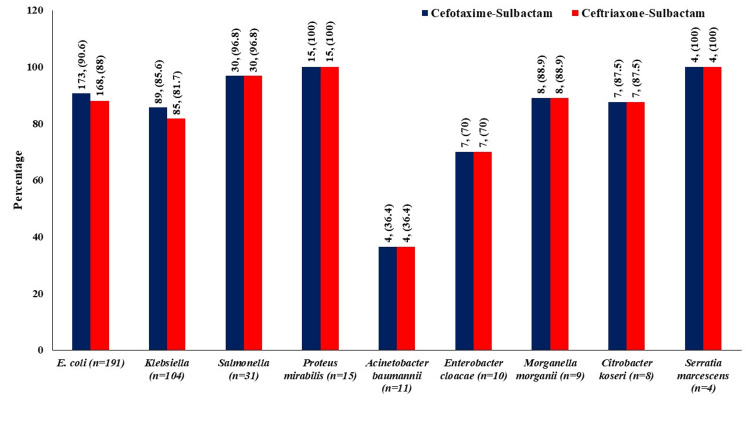
Comparison of organisms sensitive to both antibiotics as per CLSI criteria of MIC Values are in number (percentage) CLSI: Clinical and Laboratory Standards Institute; MIC: minimum inhibitory concentration

As shown in Figure 2, 173/191 *E. coli*, 89/104 *Klebsiella*, 30/31 *Salmonella*, 15/15 *Proteus*, 4/11 *Acinobacter baumanii*, 7/10 *Enterobacter cloacae*, 8/9 *Morganella morganii*, 7/8 *Citrobacter koseri, *and 4/4 *Serratia marcescens *were found susceptible to cefotaxime-sulbactam, whereas 168/191 *E. coli*, 85/104 *Klebsiella*, 30/31 *Salmonella*, 15/15 *Proteus*, 4/11 *Acinobacter baumanii*, 7/10 *Enterobacter cloacae*, 8/9 *Morganella morganii*, 7/8 *Citrobacter koseri, *and 4/4 *Serratia marcescens *were found susceptible to ceftriaxone-sulbactam as per MIC. Based on ZOI, 174/191 *E. coli*, 88/104 *Klebsiella*, 30/31 *Salmonella*, 15/15 *Proteus*, 4/11 *Acinobacter baumanii*, 7/10 *Enterobacter cloacae*, 8/9 *Morganella morganii*, 7/8 *Citrobacter koseri*, and 4/4 *Serratia marcescens *were found susceptible to cefotaxime-sulbactam, whereas 169/191 *E. coli*, 88/104 *Klebsiella*, 29/31 *Salmonella*, 15/15 *Proteus*, 4/11 *Acinobacter baumanii*, 7/10 *Enterobacter cloacae*, 8/9 *Morganella morganii*, 7/8 *Citrobacter koseri*, and 4/4 *Serratia marcescens *were found susceptible to ceftriaxone-sulbactam.

**Figure 3:**
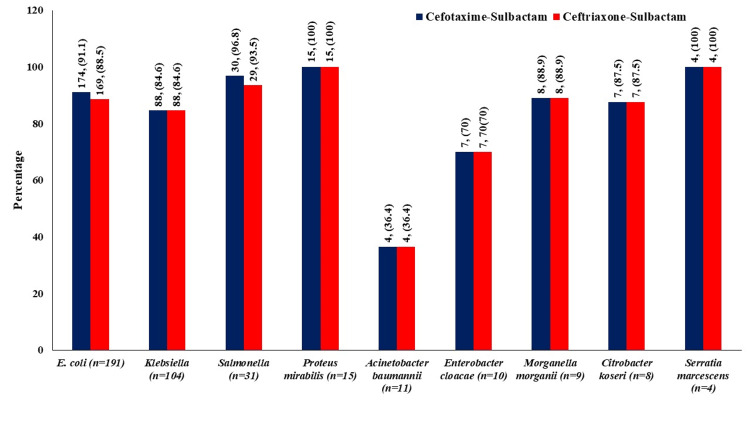
Comparison of organisms sensitive to both antibiotics as per CLSI criteria for ZOI Values are in number (percentage) CLSI: Clinical and Laboratory Standards Institute; ZOI: zone of inhibition

The proportion of susceptible organisms (based on MIC and ZOI) was found similar for *E. coli* (MIC: 90.6% vs. 88%; ZOI: 91.1% vs. 88.5%), *Klebsiella (*MIC: 85.6% vs. 81.7%), *Salmonella* (ZOI: 96.8% vs. 93.5%) and other organisms for cefotaxime-sulbactam and ceftriaxone-sulbactam (Figures 2, 3).

MIC_50_ and MIC_90 _values were also found similar for cefotaxime-sulbactam and ceftriaxone-sulbactam for all isolated organisms (Table 4).

**Table 4:** MIC50 and MIC90 values (μg/ml) of the antibiotics (cefotaxime-sulbactam and ceftriaxone-sulbactam) against different isolates

Organism	MIC_50_		MIC_90_	
	Cefotaxime-sulbactam	Ceftriaxone-sulbactam	Cefotaxime-sulbactam	Ceftriaxone-sulbactum
E. coli	0.25	0.38	1	2
Klebsiella	0.19	0.25	16	32
Salmonella	0.064	0.064	0.38	0.38
Proteus mirabilis	0.023	0.023	0.19	0.25
Acinetobacter baumannii	256	256	256	256
Staphylococcus	0.125	0.125	32	32
Enterobacter cloacae	0.38	0.38	24	24
Morganella morganii	0.064	0.064	0.5	0.5
Citrobacter koseri	0.047	0.064	4	4
Pseudomonas aeruginosa	256	256	256	256
Serratia marcescens	0.032	0.047	0.094	0.125

## Discussion

The present *in vitro* analysis evaluated the susceptibility of selected bacteria to cefotaxime-sulbactam and ceftriaxone-sulbactam isolated from clinical samples of Indian patients. Cefotaxime and ceftriaxone are β-lactam antibiotics that have a similarly broad antimicrobial spectrum and are widely used clinically for various infectious diseases. The emergence and increasing prevalence of bacterial resistance to cephalosporin antibiotics have shifted the focus of clinicians towards a combination of β-lactam and β-lactamase inhibitors, i.e., cefotaxime-sulbactam [19]. In Korea, resistance to cephalosporin was found to have increased from 1.2% (2005) to 10.2% (2010) in five years [20]. In California, 26.9% of *Enterobacteriaceae* reported in healthcare-associated infections were found to be cephalosporin-resistant [21]. The overuse of carbapenems and subsequent increase in resistance to carbapenems also demand the consideration of the role of cephalosporin-β-lactamase inhibitor combinations in overcoming bacterial resistance [22].

In an *in vitro* study by Lai et al., a 100% synergistic effect was observed after the addition of sulbactam to cefotaxime [23]. The addition of sulbactam led to the increased antimicrobial activity of β-lactam antibiotics to *Acinetobacter baumannii*, which is one of the leading infectious agents in the ICU [24]. In the present study, 36.4% of isolates of *Acinetobacter baumannii *were found susceptible to both cefotaxime-sulbactam and ceftriaxone-sulbactam. There was a significant reduction in the MIC_50_ and MIC_90_ values of various organisms like *Citrobacter freundii*, *Enterobacter cloacae*, *E. coli*, and *Klebsiella pneumoniae* when cefotaxime was combined with sulbactam [25]. Cefotaxime was found superior to ceftriaxone for minimum bactericidal concentration at which 90% of strains tested are killed (MBC_90_) values against *Streptococcus pneumoniae, Streptococcus agalactiae, Haemophilus influenzae, *and *E. coli *isolated from patients with meningitis in a study by Deguchi et al. [26]. In this study, MIC_50_ values of cefotaxime-sulbactam were found comparable to that of ceftriaxone-sulbactam (Table 4). These findings are suggestive of a comparable effect of cefotaxime-sulbactam with ceftriaxone-sulbactam in various Gram-negative and Gram-positive infections. Resistance to cefotaxime and ceftriaxone due to β-lactamase-producing organisms has become a major headache to clinicians. The frequency of resistant strains of *Klebsiella pneumoniae, Enterobacter cloacae, Serratia* *marcescens, and Acinetobacter* *baumannii* significantly (3-10 times) decreases when cefotaxime is combined with sulbactam at a 2:1 ratio compared to cefotaxime without an inhibitor [12,25]. In the present study, the proportion of organisms sensitive to cefotaxime-sulbactam was comparable to that to ceftriaxone-sulbactam (Figures 2, 3). Thus, cefotaxime-sulbactam has shown a similar *in vitro *effect to ceftriaxone-sulbactam. A comparable *in vitro* activity of cefotaxime-sulbactam against Gram-negative organisms producing β-lactamase is clinically more important for the management of various infections.

The efficacy of these combinations has been established in some clinical studies. In a study by Xin et al., the ceftriaxone/sulbactam combination eradicated 83.6% of various organisms in urinary tract infections [22]. Cefotaxime-sulbactam was found to be effective in the treatment of lower respiratory and urinary tract infections [27,28]. The high efficacy of cefotaxime/sulbactam in adults and children in the treatment of pneumonia, peritonitis, and urinary tract infections, as well as skin and soft tissues, has been shown in four clinical studies conducted in Russia and other countries [12]. In a study conducted by Makwana et al., where cefotaxime-sulbactam combination therapy was tested for the treatment of complicated urinary tract infections as compared to cefoperazone-sulbactam, a significant improvement in terms of clinical cure, microbiological cure, and late follow-up visits were found with cefotaxime-sulbactam and it was found as effective as cefoperazone-sulbactam [29]. Safety and cost become important determining factors for the selection of antibiotics when efficacy is equivalent.

Cefotaxime and ceftriaxone belong to the same class of drugs, and most of their pharmacological properties are similar. However, they differ in terms of serum protein binding, elimination half-life, and route of excretion. Ceftriaxone is more protein-bound (95% vs. 35%), has a longer elimination half-life (8.8 h vs. 1.2 h), and a more biliary excretion than cefotaxime (40% vs. 10%) [4,30]. These differences may result in differences in side effects profile also. Ceftriaxone has a higher tendency to (i) increase *Candida* species in vaginal flora, (ii) cause an overgrowth of *Candida* spp. and *Enterococci* in the intestine, and (iii) cause diarrhea, urolithiasis, and cholelithiasis as compared to cefotaxime [31-36]. There is a higher incidence of C*lostridium difficile* infections with ceftriaxone as compared to cefotaxime [37]. Ceftriaxone can cause more gut microbiota disturbances after excretion into the bile whereas cefotaxime has lesser biliary excretion than ceftriaxone. In Leipzig University Hospital in Germany, a trend of decline in the use of ceftriaxone was noted with the increased use of cefotaxime, which resulted in a sudden decrease in the incidence of *Clostridium difficile* infections [37]. Due to biliary excretion associated with ceftriaxone, *Enterobacteriaceae* harboring high-level AmpC b-lactamase (HL-CASE) increases, which in turn results in a resistant infection that increases the use of carbapenems. In a retrospective study conducted in France, it was found that the replacement of ceftriaxone with cefotaxime in clinical practice reduced and stabilized the incidence of HL-CASE *Enterobacteriaceae* [38]. Thus, the use of cefotaxime may also help in reducing the utilization of carbapenems, which are preferred in such cases. Ceftriaxone has been also found to have a strong association with immune thrombocytopenia [39]. Ceftriaxone causes serious events of immune hemolytic anemia and biliary pseudolithiasis in children, which is a major concern [40].

The main strength of the present study is that it was conducted at various centers in different regions of India with a uniform methodology. However, the number of samples included for *Proteus mirabilis, Acinetobacter baumannii*, *Staphylococcus, Enterobacter cloacae, Morganella morganii*, *Citrobacter koseri*, *Pseudomonas aeruginosa, Serratia marcescens* in this study was lower compared to the samples of *E. coli, Klebsiella, *and *Salmonella*. Infections with *Pseudomonas, Morganella morganii, Citrobacter koseri*, and *Serratia marcescens* are uncommon. A larger study with a greater number of samples of such uncommon organisms is required for drawing a proper inference about their susceptibility.

## Conclusions

The findings of the present *in vitro* study are suggestive of a comparable effect of cefotaxime-sulbactam to ceftriaxone-sulbactam on various Gram-negative clinical isolates. Thus, cefotaxime-sulbactam can be used empirically for treating various Gram-negative infections.
